# Current State of Compulsory Basic and Clinical Courses in Genetics for Medical Students at Medical Faculties in Balkan Countries With Slavic Languages

**DOI:** 10.3389/fgene.2021.793834

**Published:** 2022-01-10

**Authors:** Nina Pereza, Rifet Terzić, Dijana Plaseska-Karanfilska, Olivera Miljanović, Ivana Novaković, Željka Poslon, Saša Ostojić, Borut Peterlin

**Affiliations:** ^1^ Centre for Genetic Education, Faculty of Medicine, University of Rijeka, Rijeka, Croatia; ^2^ Department of Medical Biology and Genetics, Faculty of Medicine, University of Rijeka, Rijeka, Croatia; ^3^ Department of Biology, Faculty of Science, University of Tuzla, Tuzla, Bosnia and Herzegovina; ^4^ Research Centre for Genetic Engineering and Biotechnology “Georgi D. Efremov”, Macedonian Academy of Sciences and Arts, Skopje, North Macedonia; ^5^ Center for Medical Genetic and Immunology, Clinical Center of Montenegro, Podgorica, Montenegro; ^6^ Clinical Center of Montenegro, Medical Faculty, University of Montenegro, Podgorica, Montenegro; ^7^ Faculty of Medicine, University of Belgrade, Belgrade, Serbia; ^8^ Clinical Institute of Genomic Medicine, University Medical Centre Ljubljana, Ljubljana, Slovenia

**Keywords:** genetic education, medical genetics, human genetics, medical education, compulsory course, genomic medicine, medical students, medical faculty

## Abstract

**Introduction:** In this study we aimed to perform the first research on the current state of compulsory basic and clinical courses in genetics for medical students offered at medical faculties in six Balkan countries with Slavic languages (Bosnia and Herzegovina, Croatia, Montenegro, North Macedonia, Serbia, and Slovenia).

**Materials and Methods:** The study was conducted from June to September 2021. One representative from each country was invited to collect and interpret the data for all medical faculties in their respective country. All representatives filled a questionnaire, which consisted of two sets of questions. The first set of questions was factual and contained specific questions about medical faculties and design of compulsory courses, whereas the second set of questions was more subjective and inquired the opinion of the representatives about mandatory education in clinical medical genetics in their countries and internationally. In addition, full course syllabi were analysed for course aims, learning outcomes, course content, methods for student evaluation and literature.

**Results:** Detailed analysis was performed for a total of 22 medical faculties in Bosnia and Herzegovina (6), Croatia (4), Montenegro (1), North Macedonia (3), Serbia (6), and Slovenia (2). All but the two medical faculties in Slovenia offer either compulsory courses in basic education in human genetics (16 faculties/courses) or clinical education in medical genetics (3 faculties/courses). On the other hand, only the medical faculty in Montenegro offers both types of education, including one course in basic education in human genetics and one in clinical education in medical genetics. Most of the basic courses in human genetics have similar aims, learning outcomes and content. Conversely, clinical courses in medical genetics are similar concerning study year position, number of contact hours, ECTS (European Credit Transfer and Accumulation System) and contents, but vary considerably regarding aims, learning outcomes, ratio of types of classes, teaching methods and student evaluation.

**Conclusion:** Our results emphasise the need for future collaboration in reaching a consensus on medical genetics education in Balkan countries with Slavic languages. Further research warrants the analysis of performance of basic courses, as well as introducing clinical courses in medical genetics to higher years of study across Balkan countries.

## Introduction

Medical genetics is one of the most complex, comprehensive and multidisciplinary medical specialties covering all stages of life and organ systems, simultaneously placing a special emphasis on ethical, legal and social implications of genetic testing. Moreover, the integration of the fascinating advancements in the development of genetic and genomic testing methods into various parts of medicine occurs at an accelerated pace. Therefore, most countries in Europe, especially Western Europe, have long recognised not only the importance of introducing medical and laboratory genetics as separate medical specialties, but have also put effort into raising the level of genetic literacy among medical students as the future health professionals who will be involved in the care of patients with genetic disorders (Tobias et al., 2021).

The Balkan area is a geographical region in the south-eastern part of the European continent, associated with different cultural and historical classifications. One of these includes the classification according to the languages spoken in specific countries, such as Slavic, Romance, Turkish and other languages. Countries with Slavic languages include Bosnia and Herzegovina, Bulgaria, Croatia, Montenegro, North Macedonia, Serbia and Slovenia. In fact, these countries are not only associated by Slavic language but also similar higher education and health systems.

Unfortunately, Balkan countries with Slavic languages have encountered many historical obstacles that have left inevitable consequences in terms of significant delays in both introducing medical and laboratory genetics as medical specialties, as well as recognising genetic education at medical faculties as an indispensable tool for future physicians of the 21st century. Consequently, the advances in medical genetics internationally have not been accompanied always by an adequate level of application in clinical practice nor raising genetic literacy among medical students locally in the Balkans. Furthermore, most countries have not yet introduced medical or laboratory genetics as medical specialties, which inevitably reflects on the (poor) position of genetic education in the integrated undergraduate and graduate medical education system.

Genetic education of medical students is a critical prerequisite for appropriate care for patients with genetic disorders (Bennett et al., 2017; Hyland and Dasgupta, 2019). Because medical genetics is both a basic science and a clinical specialty, appropriate genetic education of medical students should include the literacy on basic concepts in human genetics, as well as clinical concepts in medical genetics (Robinson and Fong, 2008). However, the current situation for genetic education opportunities for medical students at medical faculties in the afore-mentioned Balkan countries is not known. Considering this, as well as the fact that Balkan countries with Slavic languages are associated by more similarities than separated by simply geographical boundaries, the aim of this study was to analyse the current state of compulsory basic and clinical courses in genetics for medical students offered at medical faculties in these countries.

## Materials and Methods

### Inclusion of Representatives From Different Balkan Countries

This retrospective study was conducted from June to September 2021. To investigate the current state of basic and clinical compulsory courses in genetics for medical students at medical faculties in Balkan countries in which Slavic languages are spoken, the study was designed so that one representative from each of the selected countries was invited to collect and interpret the data for all medical faculties in their respective country.

An additional four representatives from four different Balkan countries with Slavic languages (Bosnia and Herzegovina, Montenegro, North Macedonia, and Serbia) were contacted *via* e-mail in June 2021 with a letter of invitation to participate in the study. The representatives were chosen based on their expertise, as well as national and international excellence in the field of both basic human genetics and clinical medical genetics. The letter of invitation contained all the relevant information regarding the research, including an explanation of the background, aims, materials and methods. In addition, in this invitation letter, the representatives were sent and asked to fill a questionnaire about the basic and clinical compulsory courses in genetics offered in their respective countries at medical faculties for medical students and a due date was provided. All six representatives (Bosnia and Herzegovina, Croatia, Montenegro, North Macedonia, Serbia and Slovenia) filled the questionnaire and were sent a second e-mail with the request to send the full syllabi for each course mentioned in the questionnaire. The second e-mail also contained a detailed explanation of the reasons for requesting the full course syllabi (evaluation of course aims, learning outcomes, course content, methods for student evaluation and mandatory literature).

All representatives participated in the research voluntarily. Considering that this research is a retrospective study, no approval of ethical committees was necessary.

### Questionnaire

A short questionnaire was designed with the aim of collecting the relevant data about basic and clinical compulsory courses in genetics for medical students at medical faculties in Balkan countries in a concise and uniform manner. The questionnaire consisted of two sets of questions.

The first set of questions was factual and contained specific questions about mandatory education, including the names of the medical faculties in their respective countries and titles of basic and clinical compulsory courses in genetics offered at each medical faculty for medical students. In addition, for each course, the representatives were asked to grade the appropriateness of the study years on which the courses are offered at each faculty (level too low/appropriate/too high), number of contact hours (insufficient/appropriate/too high), and ECTS (underestimated/appropriate/overestimated).

The second set of questions was more subjective and inquired the opinion of the representatives about mandatory education in clinical medical genetics in their countries and internationally. The questions were: “Do you think that there should be a single, uniform curriculum for all compulsory courses in medical genetics in your country?”, “Do you think that there should be a single, uniform curriculum for all compulsory courses in medical genetics internationally?”, “Is medical genetics recognized as a medical specialty in your country? If yes, from which year”, “Is laboratory genetics recognized as a medical specialty in your country? If yes, from which year”, and “What are the main obstacles for optimization of the courses in your country?”.

### Full Course Syllabi

Data extracted, analysed and compared from full course syllabi were course aims, learning outcomes, course content, methods for student evaluation and literature.

## Results

Representatives of six Balkan countries with Slavic languages (Bosnia and Herzegovina, Croatia, Montenegro, North Macedonia, Serbia, and Slovenia) participated in the research. Detailed analysis was performed for the total number of medical faculties in these countries, which is 22 (Bosnia and Herzegovina 6, Croatia 4, Montenegro 1, North Macedonia 3, Serbia 6, Slovenia 2). All but two medical faculties (Faculty of medicine, Universities of Ljubljana and Maribor in Slovenia) offer either compulsory courses in basic education in human genetics (16 faculties/courses) or clinical education in medical genetics (3 faculties/courses). On the other hand, only one medical faculty offers both types of education, including one course in basic education in human genetics and one in clinical education in medical genetics (Faculty of Medicine, University of Montenegro, Podgorica). Data on the 20 medical faculties that offer compulsory courses in genetics for medical students is shown in Tables 1, 2.

**TABLE 1:** Basic courses in genetics offered at medical faculties for medical students in Balkan countries with Slavic languages.

Country	Names of medical faculties in country	Titles of the compulsory courses offered at each medical faculty	Number of contact hours in course	Number of ECTS for the course	Study year at which the course is offered
Bosnia and Herzegovina	Faculty of Medicine, University of Banja Luka	Human Genetics	75	6	1st
	Faculty of Medicine Foca, University of East Sarajevo	Cell Biology and Human Genetics	135	9	1st
	Faculty of Medicine, University of Sarajevo	Cell Biology and Human Genetics	75	6	1st
	Faculty of Medicine, University of Tuzla	Biology with Human Genetics	75	7	1st
	Faculty of Medicine, University of Zenica	Medical Biology with Human Genetics	50	5	1st
	School of Medicine, University of Mostar	Medical Genetics	45	4	2nd
Croatia	Faculty of Medicine, University of Split	Immunology and Medical Genetics	95	6	2nd
Montenegro	Faculty of Medicine, University of Montenegro, Podgorica	Human genetics	90	6	1st
North Macedonia	Faculty of Medicine, SS. Cyril and Methodius University, Skopje	Human genetics	60	5	1st
	Faculty of Medical Sciences, Goce Delcev University, Stip	Human genetics	45	4	1st
	Faculty of Medical Sciences, State University, Tetovo^a^	Human genetics	45	4	1st
Serbia	Faculty of Medicine, University of Belgrade	Human Genetics	75	6	1st
	Faculty of Medical Sciences, University of Kragujevac	Human Genetics	60	6	1st
	Faculty of Medicine, University of Novi Sad	Biology with Human Genetics	75	8	1st
	Faculty of Medicine, University of Niš	Molecular and Human Genetics	75	7	1st
	Faculty of Medical Sciences, University of Prishtina^b^	Human Genetics	75	7	1st
	Medical Faculty of the Military Medical Academy, University of Defence in Belgrade	Human Genetics	75	7	1st

a Teaching in performed in Albanian language.

b Temporary headquarers in Kosovska Mitrovica.

**TABLE 2:** Clinical courses in medical genetics offered at medical faculties for medical students in Balkan countries with Slavic languages.

Country	Names of medical faculties in country	Titles of the compulsory courses offered at each medical faculty	Number of contact hours in course	Number of ECTS for the course	Study year at which the course is offered
Croatia	Faculty of Medicine, University of Zagreb	Medical Genetics	45	4	6
	Faculty of Medicine, University of Rijeka	Medical Genetics	45	3	5
	Faculty of Medicine, University of Osijek	Medical Genetics	45	4	6
Montenegro	Faculty of Medicine, University of Montenegro, Podgorica	Clinical genetics	60	4	5

### Basic Courses in Human Genetics

#### General Features

A total of 17 compulsory basic courses in human genetics are offered at 17 medical faculties in five countries (Bosnia and Herzegovina 6, Croatia 1, Montenegro 1, North Macedonia 3, Serbia 6) (Table 1). While most courses are similar according to their position in the study years (15 in the first year, two in the second year), the courses vary considerably regarding the number of contact hours (45–135) and ECTS (4–9). Furthermore, the representative of Croatia stated that a small percentage of the compulsory course “Medical biology”, which is offered on all four medical faculties in the country, is dedicated to the basics of human genetics but this is not reflected in the title of the courses and is therefore not presented in Table 1.

Representatives of Bosnia and Herzegovina, Montenegro and North Macedonia agree that the position of the courses regarding the study years is too low. On the contrary, the representative of Serbia considers that the positions for the basic courses are appropriate in their country but emphasises the importance of introducing additional mandatory education in clinical genetics in the later study years. A special emphasis should be placed on the Faculty of Medicine, University of Split (Croatia), where the title of the basic course “Immunology and Medical Genetics” does not reflect its content, which is a mixture of both basic and clinical topics.

In addition, representatives of Bosnia and Herzegovina (regarding medical faculties in Banja Luka, East Sarajevo and Mostar), Croatia, Montenegro and Serbia agree that the number of contact hours and ECTS is appropriate for the respective courses. On the other hand, the representatives of Bosnia and Herzegovina (regarding medical faculties in Sarajevo, Tuzla, Zenica and Mostar) and North Macedonia state that the number of contact hours and ECTS in insufficient.

### Analysis of Full Course Syllabi

The analysis of full course syllabi across different Balkan countries (indicated in Table 1) revealed many similarities with only a few differences, which can be attributed to the freedom of each course coordinator, as well as specificities of the faculties’ full curricula. The only exception is the course “Immunology and Medical Genetics” at the Faculty of Medicine, University of Split (Croatia), which contains mostly basic topics with a hint of practical topics, and a consequently unclear aim and learning outcomes of the course and was therefore excluded from further comparison. Also, the title of the course “Medical genetics” at the Faculty of Medicine, University of Mostar (Bosnia and Herzegovina) would correspond more to a “Human Genetics” type of course according to their aims, learning outcomes and contents. The mandatory literature is similar for all courses (Cooper, 2000), and, additionally, at certain medical faculties, the course coordinators have their own accredited handbooks.

The aims were highly similar between courses, and mostly referred to the basic principles of modern biology and genetics (e.g. cell, biology, molecular biology, developmental biology and human genetics), focusing on the important molecular mechanisms that are important to human health, as well as the diagnosis and therapy of human diseases. Furthermore, learning outcomes were also comparable regarding knowledge, skills, and attitudes, although the biggest differences can be attributed to the level of performance required from the student. Moreover, the course content is again similar with certain specificities; however, the topics are relevant for medical students and up to date for the field of modern human genetics. The topics cover a wide array of content, from the structure of nucleic acids and chromosomes to the basics of genetic disorders aetiology (e.g. gene mutations, chromosome aberrations, epigenetic modifications) and modern methods for detection of genetic disorders. Finally, the biggest differences are present in the methods for student evaluation, especially in terms of grading and number of tests used. Although student evaluation is based mostly on the assessment of knowledge, some courses use only written exams, whereas others use both written and oral exams. With a few exceptions, the acquisition of skills is not assessed in most courses, i.e., assessment does not reflect the expected learning outcomes regarding skills.

### Clinical Courses in Medical Genetics

#### General Features

A total of four compulsory basic courses in medical genetics are offered in two countries (Croatia—Faculties of Medicine, University in Rijeka, Osijek and Zagreb, and Montenegro—Faculty of Medicine, University in Podgorica) (Table 2). Two of the courses are offered at the fifth year and two at the sixth year of study. All four studies are similar according to the number of contact hours (45–60) and ECTS (3–4).

All representatives agree that the position of the respective courses in the study year is appropriate. On the other hand, the representative of Montenegro stated that the number of contact hours and ECTS in insufficient for their course, whereas the representative of Croatia agrees that it is appropriate.

Finally, an additional course offering mandatory education in clinical genetics is integrated with pediatrics at the Faculty of medicine, University in Maribor (Slovenia). However, the program is focused only on genetics in the paediatric period and was therefore excluded from further analysis. In addition, in Slovenia at the Faculty of Medicine, University of Ljubljana some of the medical genetic topics are included in other basic or clinical courses.

#### Analysis of Full Course Syllabi

Unlike the basic courses in human genetics, the four clinical mandatory courses in medical genetics (Table 2) are similar only regarding the course contents, whereas they vary considerably with respect to the aims, learning outcomes, types of classes, ratio of types of classes, teaching methods and methods for student evaluation. The mandatory literature for the courses offered at the medical faculties of Zagreb, Osijek and Podgorica is the same (Turnpenny and Ellard, 2012), whereas the course “Medical Genetics” offered at the Faculty of Medicine, University of Rijeka has its own accredited mandatory literature.

The course “Medical Genetics” offered at the Faculty of Medicine, University of Rijeka (Croatia) consists of 17 h of lectures, 15 h of seminars and 13 h of practicals. The entire course is conducted exclusively through active learning methods and is designed and performed through case-based reasoning, thus achieving both clinical reasoning and a simulation of the actual physician-patient relationship in practice. The learning outcomes were determined and derived in accordance with key competencies according to Core Competences in Genetics for Health Professionals in Europe published by the European Society of Human Genetics specifically for physicians who are not specialists in medical genetics (ESHG European Society of Human Genetics, 2008; Čargonja et al., 2021). The final exam is delivered in the form of patient management problems, evaluating knowledge, skills, and attitudes at the same time.

The course “Medical Genetics” offered at the Faculty of Medicine, University of Zagreb (Croatia) consists of 20 h of lectures, 5 h of seminars and 20 h of practicals. Practicals are conducted at the clinics for pediatrics and the final exam is a written test. On the other hand, the third course delivered in Croatia, “Medical Genetics” at the Faculty of Medicine, University of Osijek (Croatia) consists of 27 h of lectures and 18 h of seminars.

Finally, the course “Clinical Genetics”, which is delivered at the Faculty of Medicine, University of Podgorica (Montenegro) resembles the course “Medical Genetics” at the Faculty of Medicine, University of Rijeka regarding the aim and learning outcomes, although it has more contact hours, thus enabling a wider approach in topics. The final exam consists of the practical and oral part.

### Reflections on Uniform Curricula Locally and Internationally

The representatives of all six countries agree that there should be a single, uniform curriculum for all compulsory courses in medical genetics in their respective countries. The representative of Bosnia and Herzegovina believes that it would allow easier cooperation and coordination of program. However, the representatives of Croatia and Slovenia believe that although a common framework would be helpful, some variations and freedom should be allowed between faculties due to specificities in medical genetics practice in each country and curricula of other subjects. The representative of Croatia emphasises that this curriculum should not be provisory but should also be aligned with the already existing document Core Competences in Genetics for Health Professionals in Europe published by the European Society of Human Genetics specifically for physicians who are not specialists in medical genetics (ESHG).

The representatives demonstrated more variation in their answers to the question on whether a there should be a single, uniform curriculum for all compulsory courses in medical genetics internationally. For example, the representatives of Croatia, Montenegro and Serbia think that a common framework for the Balkan area would be more appropriate due to the local specificities and different level of genetic services. On the contrary, the representatives of North Macedonia and Slovenia believe that there should be a common framework, although adapted to national health systems, which would enable common standards of knowledge for the European Union health systems, whereas the representative of Bosnia and Herzegovina thinks that a single uniform curriculum for all compulsory courses internationally would lead to better optimization of the scientific plan. Finally, all representatives agree that variations and freedom should be allowed to each course coordinator.

### Opportunities for Training in Medical and Laboratory Genetics in Balkan Countries

#### Medical Genetics as a Medical Specialty

Of the six included countries, medical genetics is offered as a medical specialty only in North Macedonia (from 2015) and Slovenia (from 2002). In Montenegro and Serbia, clinical genetics is offered as a sub-specialist education after a previously completed specialty (e.g. in pediatrics, internal medicine, gynaecology, etc.). Neither of the previously mentioned opportunities are offered in Bosnia and Herzegovina and Croatia.

#### Laboratory Genetics as a Medical Specialty

Similar to the opportunities for medical genetics training, laboratory genetics is available as a medical specialty in North Macedonia and Slovenia. In the case of North Macedonia, training in medical genetics was previously available only for biologists at the Medical faculty, University in Skopje; however, a new specialty—Clinical laboratory genetics, was introduced in 2012, which is open to health professionals, including medical doctors. In Montenegro, training in laboratory genetics is recognized in terms of the necessary conditions for work in genetic laboratories but residents need to perform their training in other countries considering that it is not available in their country. Neither of the previously mentioned opportunities are offered in Bosnia and Herzegovina and Croatia.

### Obstacles for Optimization of Clinical Courses in Medical Genetics in Balkan Countries

In the final question, the representatives were asked to share their opinion on the main obstacles for optimization of the courses in their respective countries.

The representative of Bosnia and Herzegovina shared a detailed evaluation on the current situation in their country, including that knowledge of medical genetics among teaching staff is very limited considering that there are no specialists in medical and laboratory genetics. In addition, financial challenges are obvious, especially in organizing laboratory work, such as demonstrations. Finally, the representative emphasises the inconsistencies of the entire education system as a separate issue.

The representative of Croatia believes that the fact that mandatory clinical courses in medical genetics are even offered in Croatia is a success of its own considering there is no training in medical or laboratory genetics. The biggest issue for their optimization is the lack of sufficient awareness of clinical decision makers about the importance of medical genetics and its place in modern medicine, which contrasts with great agility among medical faculty teachers towards the introduction of medical genetics in clinical practice, especially at the Faculty of Medicine, University of Rijeka. The fact that clinicians underestimate the necessity that medical students learn about medical genetics and do not integrate genetic contents or discuss patients with genetic disorders with their students represents the greatest obstacle for proper implementation of medical genetics in clinical practice in Croatia. One of the possible reasons for this is the low level of genetic literacy among different specialists. The representative of Montenegro, who believes that the small population of the country does not enable the sustainability of all types of education and that there is insufficient awareness of decision makers about the importance of medical genetics and its place in modern medicine, shared a similar opinion. In addition, the representative of Serbia thinks that better synchronization is needed between basic, laboratory and clinical aspects of medical genetics, both in education and in practice in their country. Finally, the Slovenian representative believes that there is a disconnection between medical faculties, which are dominated by non-medical scientists involved in teaching and decision making, and clinical centres, which are the seats of actual genetic medical practice.

## Discussion

In the present study, we evaluated the current state of compulsory basic and clinical courses in genetics for medical students offered at medical faculties in six countries associated by Slavic languages, including Bosnia and Herzegovina, Croatia, Montenegro, North Macedonia, Serbia, and Slovenia. With the help of representative authorities in both human and medical genetics from each country, we performed the first such study in the Balkan peninsula, which was of the utmost importance for gaining insight into the present situation, as well as planning for future directions in mandatory genetics education at medical faculties for medical students in this area. A detailed analysis of each country revealed that Bosnia and Herzegovina and Serbia precede in the number of medical faculties (six in each country), and are followed by Croatia (4), North Macedonia (3), Slovenia (2), and Montenegro (1). Except for Slovenia, all other countries offer some sort of compulsory courses in genetics for medical students: either courses in basic education in human genetics (Bosnia and Herzegovina, North Macedonia, Serbia) or both basic education in human genetics and clinical education in medical genetics (Croatia and Montenegro). However, in the case of Croatia, basic education in human genetics is offered at just one medical faculty, whereas clinical education in medical genetics is offered at three different medical faculties. Therefore, currently the best example for an integrative approach to medical students’ comprehensive education in genetics is represented by the Faculty of Medicine, University of Podgorica in Montenegro, which offers basic education in human genetics in the first year of study and clinical education in medical genetics at the fifth year of study.

### Basic Courses in Human Genetics

Compulsory basic courses in human genetics are offered at 17 medical faculties in five countries (Bosnia and Herzegovina 6, Croatia 1, Montenegro 1, North Macedonia 3, Serbia 6). Interestingly, except for Croatia, which represents a special case, and Slovenia, which does not offer any type of basic education in human genetics, this result indicates that mandatory education in human genetics is offered at every medical faculty in Bosnia and Herzegovina, Montenegro, North Macedonia, and Serbia. Most of the courses (15) are offered in the first year of study, with highly similar aims, learning outcomes and course content. Although the mandatory literature is also similar, commendably, certain course coordinators also have their own accredited handbooks, emphasising and encouraging the importance of allowing freedom to each course coordinator. All these results indicate high awareness of the importance of basic sciences in modern medicine in these countries and represents an excellent basis for the introduction of clinical courses in medical genetics in the later years of study, like in Montenegro.

As indicated, Croatia represents a special case because although a compulsory course “Medical biology” is offered at all four medical faculties in the country, covering certain topics of the basics of human genetics, this is not reflected in the title of the course and was therefore excluded from further analysis. However, an initiative might be launched at the national level to rename the courses to reflect their contents in a more accurate manner (e.g. Medical biology with human genetics). We also encountered certain illogicality at the Faculty of Medicine, University of Split, where the title of the basic course “Immunology and Medical Genetics” does not reflect the content and should therefore be renamed and separated from immunology. In addition, after the modification of the course aims, learning outcomes and contents, the course should be moved to a higher year of study, as is the case with the remainder of medical faculties in the country. It is unclear how this artificial merging of two highly diverse courses occurred considering that this not in line with the Croatian national curriculum.

Although the representatives of Bosnia and Herzegovina, Montenegro and North Macedonia believe that the position of the courses are too low in the study year, the representative of Serbia considers that the position is appropriate and that an additional clinical course should be introduced at the higher years of study.

### Clinical Courses in Medical Genetics

The current situation regarding compulsory clinical courses in medical genetics is completely different than for basic courses in human genetics. Generally, clinical courses in medical genetics are highly underrepresented in Balkan countries. Specifically, only four compulsory clinical courses are offered in just two countries—at three medical faculties in Croatia and one in Montenegro. Interestingly, neither country offers medical or laboratory genetics as a medical specialty. In addition, although these courses are similar with regards to study year position (fifth or sixth year), number of contact hours (45–60), ECTS (3–4) and contents, they vary considerably with respect to the aims, learning outcomes, types of classes, ratio of types of classes, teaching methods and methods for student evaluation. Not only do the courses vary between Croatia and Montenegro, but they also vary substantially between the medical faculties in Croatia. For example, students attend practicals only at the pediatrics departments at the Faculty of medicine, University in Zagreb, whereas at the Faculty of medicine, University of Osijek, students do not have practicals at all. On the other hand, at the Faculty of medicine, University of Rijeka, the course is based on clinical reasoning and is aligned with key competencies according to Core Competences in Genetics for Health Professionals in Europe published by the European Society of Human Genetics specifically for physicians who are not specialists in medical genetics (ESHG European Society of Human Genetics, 2008; Robinson and Fong, 2008). The course content, teaching methods (primarily case-based reasoning) and methods of evaluation were analysed in detail on two generations of medical students and the results, which were previously published (Čargonja et al., 2021), confirmed that needs-based education not only increases the knowledge of medical students, but also helps develop positive attitudes and self-confidence, which is crucial for proper patient care. It is noteworthy to emphasise that the same course at the same medical faculty was among the most problematic in the entire medical study several years ago and received constant negative feedback from students. The main reason for this criticism from students was highly justified since the course contained mostly basic topics in human and laboratory genetics, such as detailed descriptions of methodology and even performance of molecular-genetic methods of genetic testing, which is not relevant for future physicians. All of this is in line with the adult learning theory, in which motivation and purposefulness of content is crucial (Thammasitboon and Brand, 2021). However, the course was completely altered with the new course coordinator and is now in tune with the actual requirements of medical professionals at the end of their integrated undergraduate and graduate education.

### Obstacles for Optimization of Clinical Courses in Medical Genetics in Balkan Countries

The reasons for such low integration of compulsory clinical courses in medical genetics at the medical faculties for medical students in Balkan countries are numerous. The Balkan area is a highly specific geographic area in Southeast Europe and is sometimes associated with different cultural and historical explanations. First, this is an area which is synonymous with conflict and violent confrontation, which undoubtedly slowed down the progress and development of certain Balkan countries. The best evidence for this is Slovenia, which is the only country that did not suffer substantial war consequences and was the first of the Balkan countries included in this study to introduce both medical and laboratory genetics specialties and experience profound progress in the application of the most modern technologies in genetic testing to everyday clinical practice. In fact, specialists in medical and laboratory geneticists from Slovenia are the ones who are nowadays helping professionals in other Balkan countries develop medical and laboratory genetics with their knowledge, experience, and clinical services. Second, a direct consequence of the afore-mentioned concerns are economic issues of the Balkan countries, which are still evident in the present time (emphasised by the representatives of Bosnia and Herzegovina and Croatia) and does not allow for the same opportunities for the procurement of expensive modern genomic technologies as in the Central and West European countries. Third and final, considering the substantial delay in medical genetics in comparison with West European countries, most diagnostic genetic laboratories were led by non-medical professionals, especially biologists and molecular biologists, who were consequently also the first course coordinators of clinical courses in medical genetics (especially in Croatia). Considering that non-medical professionals did not associate the contents in their courses with clinical practice, future physicians did not see the benefits of medical genetics in clinical practice. When these students became physicians, they could not integrate medical genetics into their clinical teachings, leading to a consequently huge gap and a vicious circle between basic scientists and clinicians, which is still ongoing.

In this study, the representative of each country shared their opinion on this topic for their country and these are in line with the afore-mentioned issues. With certain specificities in their answers, all representatives agree that the biggest issue in each country is insufficient awareness of decision makers (be they clinical or basic professionals) about the importance of medical genetics and its place in modern medicine.

### Directions for the Future

In terms of the basic courses in human genetics, although they are highly similar on paper (with respect to biggest differences in the methods for student evaluation, as expected), further research would require the analysis of course performance. Therefore, future research would require peer-review and attendance of all courses to evaluate the transfer of content to students, especially in the context of analysing the achievement of course aims and learning outcomes, as well as applied teaching and learning methods (e.g. the application of active learning methods and better horizontal integration with clinical courses). Future studies should also analyse vertical integration with clinical courses to allow for updates in the curricula. Also, feedback from student evaluation of the courses must be considered because student opinion is crucial for advancing any curriculum or syllabus.

For the clinical courses, Balkan countries are in desperate need of introducing these to higher years of study consequent to the rapid development of medical genetics and its integration into all fields of modern medicine. However, course coordinators should bear in mind that it is crucial that their courses are aligned with the minimum core competencies for future physicians and that the education is needs-based. Otherwise, if medical students do not see usefulness, purposefulness, and application of the course contents in their future clinical practice, opposite, unwanted effects might be achieved. Therefore, it would be important to follow the rules of adult-learning theory and apply active learning methods (e.g. clinical reasoning) and critical thinking to the maximum extent (Wolyniak et al., 2015; Čargonja et al., 2021). Although representatives of all six countries agree that a consensus in the form of a national and/or regional Balkan curriculum might benefit medical faculties, it is important to allow freedom to each course coordinator to align the course with national and local specificities.

Additionally, vertical and horizontal integration of medical genetics with other clinical courses would be of the utmost importance and continuous emphasis on the importance of genetics through other medical specialties to medical students is indispensable for their understanding of the importance genetics has in modern medicine. Thus, genetic education of clinicians of other specialties might help prevail this obstacle.

Finally, only two countries offer medical and laboratory genetics as a medical specialty (North Macedonia and Slovenia), and in addition to introducing mandatory genetic education for medical students and clinicians, the remaining countries should focus on the introduction of both specialties for postgraduate students.

## Conclusion

In the present study, we performed the first research on the current state of basic and clinical courses in genetics for medical students offered at medical faculties in six Balkan countries with Slavic languages (Bosnia and Herzegovina, Croatia, Montenegro, North Macedonia, Serbia, and Slovenia). Except for Slovenia, all other countries offer some sort of compulsory courses in genetics for medical students at a total of 20 medical faculties: either courses in basic education in human genetics (Bosnia and Herzegovina, North Macedonia, Serbia) or both basic education in human genetics and clinical education in medical genetics (Croatia and Montenegro). Most of the basic courses in human genetics are similar concerning their aims, learning outcomes and course content. On the other hand, clinical courses in medical genetics are offered only at three medical faculties in Croatia and one in Montenegro. In addition, although these courses are similar with regards to study year position, number of contact hours, ECTS and contents, they vary considerably with respect to the aims, learning outcomes, ratio of types of classes, teaching methods and student evaluation. Further research warrants the analysis of performance of basic courses, as well as introducing clinical courses in medical genetics to higher years of study across Balkan countries. Increasing genetic literacy in medical genetics in clinicians of other medical specialties is also crucial. Finally, this study emphasises the need for collaboration and is the first step towards breaking the years-long barriers that have prevented the consensus on medical genetics education in Balkan countries with Slavic languages, all for the benefit of future physicians and their patients.

## Data Availability

The raw data supporting the conclusions of this article will be made available by the authors, without undue reservation.
